# An Experimental Study of Micro-Dimpled Texture in Friction Control under Dry and Lubricated Conditions

**DOI:** 10.3390/mi13010070

**Published:** 2021-12-31

**Authors:** Yuan Wei, Jesus Resendiz, Robert Tomkowski, Xu Liu

**Affiliations:** 1 Frontiers Science Center for Flexible Electronics, Xi’an Institute of Flexible Electronics (IFE) and Xi’an Institute of Biomedical Materials & Engineering, Northwestern Polytechnical University, Xi’an 710072, China; 2 Manufacturing and Metrology Systems, KTH Royal Institute of Technology, 10044 Stockholm, Sweden; rtom@kth.se; 3 Department of Mechanical and Manufacturing Engineering, University of Calgary, Calgary, AB T2N 1N4, Canada; jdjresen@ucalgary.ca; 4 School of Mechanical Engineering, Xi’an Aeronautical University, Xi’an 710077, China

**Keywords:** laser surface texturing, micro dimples, dry friction, friction reduction

## Abstract

Friction control is a vital technology for reaching sustainable development goals, and surface texturing is one of the most effective and efficient techniques for friction reduction. This study investigated the performance of a micro-dimpled texture under varying texture densities and experimental conditions. Reciprocating sliding tests were performed to evaluate the effects of the micro-dimpled texture on friction reduction under different normal loads and lubrication conditions. The results suggested that a micro-dimpled texture could reduce the coefficient of friction (CoF) under dry and lubricated conditions, and high dimple density results in a lower CoF. The dominant mechanism of the micro-dimpled texture’s effect on friction reduction was discussed, and surface observation and simulation suggested that a micro-dimpled texture could reduce the contact area at the friction interface, thereby reducing CoF.

## 1. Introduction

Energy and material loss due to tribological contacts significantly affect energy consumption globally. Around 23% of the world’s total energy consumption is used to overcome friction and to remanufacture contacting parts because of material loss [1]. Thus, minimizing friction in mechanical systems is vital for saving energy and reducing waste in our society. Using surface texture to reduce friction has become a focus of worldwide research. For decades, researchers have demonstrated that certain micro-textures minimize friction and wear. Several studies indicated that surface texture decreases the friction coefficient [2,3] and improves the lubrication condition of friction pairs by enhancing the hydrodynamic pressure effect [4]. Surface texturing techniques have been widely applied in several areas, such as cutting tools [5], bearings [6], cylinders [7], pistons [8], and rotating shafts [9].

A variety of techniques have been employed to fabricate surface texturing, including machine processing, chemical etching, electrical discharge machining, energy beam etching, and laser surface texture (LST). Laser surface texturing is a highly efficient, precise, versatile, and economical method to fabricate micro dimples and micro grooves compared to other texturing processes [10]. To date, significant improvements in surface texturing have been made in the past few decades. During the 1990s, Etsion et al. presented laser-textured spherical dimples on seal surfaces, which could theoretically enhance lubrication conditions [11], and the performance of the spherical dimple texture was verified experimentally the following year [12]. Pettersson et al. investigated the micro grooves under dry and lubricated conditions, and, under certain conditions, friction was reduced [13]. Numerous studies on surface texture have been published more recently. Wan et al. [14] reported that the coefficient of friction (CoF) was reduced by 80.1% due to a micro-dimpled texture under lubricated conditions, and Hao et al. found that friction decreased by 46.1% with linear grooves under lubricated conditions [15]. However, several studies reported an opposite effect; Hu et al. found that the CoF increased by 400% with circular dimples on a steel surface [16], and Dai et al. reported a 3.6% increase in CoF with micro-dimple textured Ti-alloy surfaces [17]. Therefore, the conflicting reports in CoF trends in the literature indicate the uncertainty of the performance of LST texture, which is a challenge for further applications of LST texturing. Texture effects greatly rely on shape [18,19,20], size [21,22], and depth [23]. Other factors include density [24,25], area [26], and orientation [27] of the texture.

In general, surface texture can positively affect sliding surfaces, such as storing oil, catching debris, creating a hydrodynamic, pushing-up force, and reducing the contact area [28]. The performance of a micro-dimpled texture is determined by the combined effect of those mechanisms. However, under different solid contact conditions at the friction interface, the dominant mechanism changes. For instance, under hydrodynamic lubrication, which has no solid contact at the friction interface, surface texture can lead to an increase in load carrying capacity of the lubricant film [4]. Thus, lubrication conditions could be the main factor that determines the performance of surface textures. Consequently, more research is needed in the area of friction control using micro dimples under varying lubrication conditions.

In this study, the effect of micro-dimpled texture is investigated using reciprocating sliding tests under dry and lubricated conditions and varying normal loads. A pulsed laser with interval power output is used to fabricate the micro-dimpled texture. Experimental results suggested that the micro-dimpled texture significantly decreased CoF under both dry and lubricated conditions. The density of the micro-dimpled texture was proportional to the degree of friction reduction for all tested conditions. Furthermore, the mechanism of the micro-dimpled texture effect was discussed.

## 2. Texture Preparation

**Figure 1:**
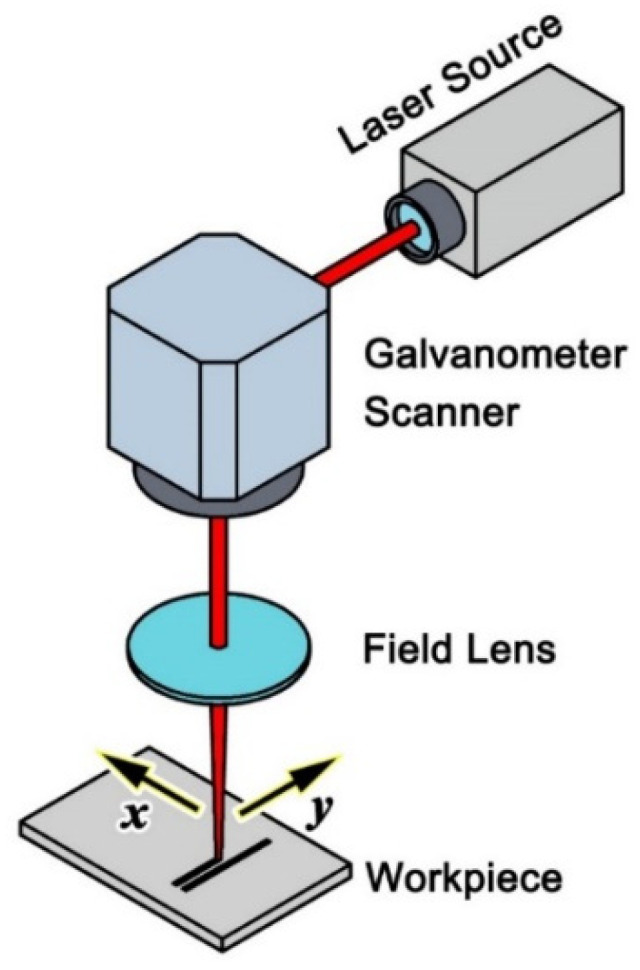
Schematic diagram of the laser marking system.

In this study, the micro-dimpled texture was produced using a commercial laser marking system. The schematic diagram of the system is shown in Figure 1, and the specifications are listed in Table 1. A laser beam passed through a galvanometer scanner and a field lens and then irradiated the workpiece surface directly. The scanner could quickly adjust the position of the laser beam using a two-axis mirror galvanometer system. The laser beam had a nearly Gaussian profile (>95%).

AISI/ASTM 52100 bearing steel was selected as the workpiece material. The workpiece was machined as a cuboid with dimensions of 30 mm × 15 mm × 5 mm. The contact surfaces, including the textured surface attached to the friction tester stage, were polished. The average surface roughness for the polished surfaces was *S_a_* = 0.2 μm and *S_q_* = 0.3 μm. Heat treating was not performed on the workpieces. Micro-dimpled textures were fabricated on the workpiece surfaces, and the textured area was 450 mm^2^. During the texturing process, the laser output was set at 8 W, the spot moving speed was 2000 mm/s, and the pulse frequency was 20 kHz.

**Figure 2:**
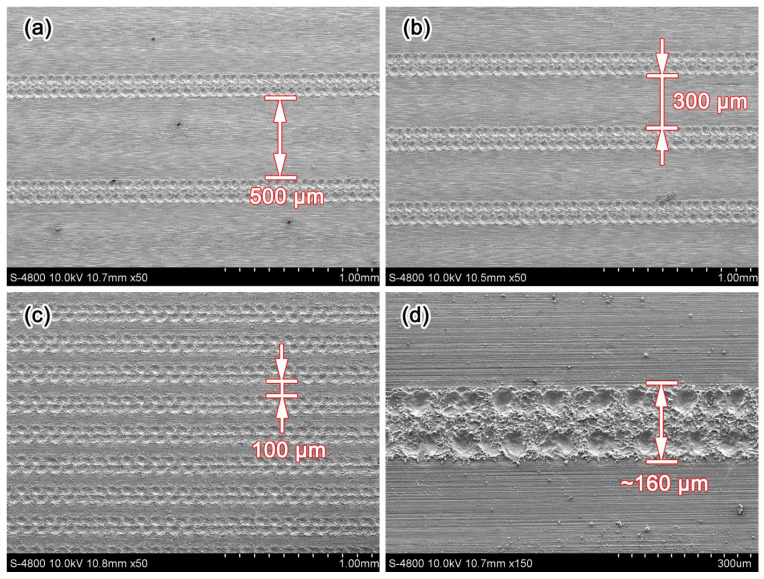
SEM images of the textured surfaces for dimple densities of (**a**) 25.3%, (**b**) 36.2%, (**c**) 63.0%, and (**d**) partially enlarged dimples of 63.0%.

The textured surfaces were observed using scanning electron microscopy (SEM), as shown in Figure 2. The micro dimples were arranged tightly in rows rather than in a uniform distribution across the workpiece surface, and two rows of micro dimples formed a textured belt. Three different textures were prepared, each with a different distance between the textured belt: 500 μm, 300 μm, and 100 μm, and the dimple density for each were (a) 25.3% low density, (b) 36.2% medium density, and (c) 63.0% high density, respectively.

## 3. Reciprocating Sliding Test

**Figure 3:**
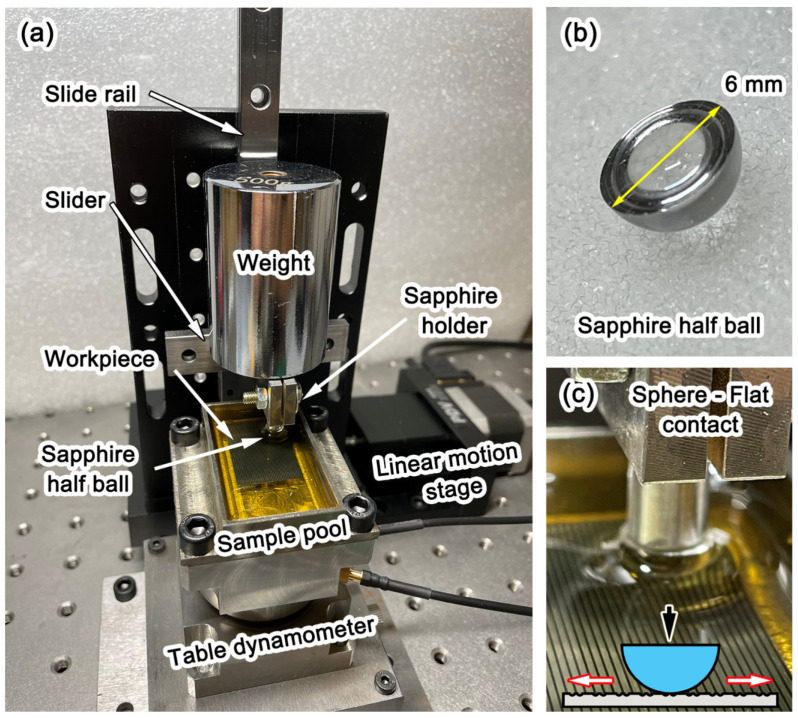
Experimental setup of the reciprocating sliding tests: (**a**) homemade reciprocating sliding friction test platform; (**b**) sapphire half ball; (**c**) sphere-flat contact.

The effect of the micro-dimpled texture in tribological contacts has been evaluated using reciprocating sliding tests under both dry and lubricated conditions. The tests were performed using a homemade reciprocating friction tester. The experimental setup is shown in Figure 3. The workpiece was mounted on a three-component quartz table dynamometer. The force signals were collected by a data-acquisition system and recorded in a computer with a sampling rate of 1024 Hz. The sliding motion was driven by a CNC linear motion stage. Sapphire (Al_2_O_3_) was selected as the other material in the friction pair, and a 6 mm-diameter sapphire half ball was mounted on the tester. The properties of sapphire are shown in Table 2. For the lubricated tests, SAE 0W30 engine oil was selected as the lubricant. The initial lubricant temperature was room temperature (20 °C), and 20 cm^3^ of engine oil was added to the sample pool (as shown in Figure 3c) before each test. The workpiece was immersed in the engine oil.

**Figure 4:**
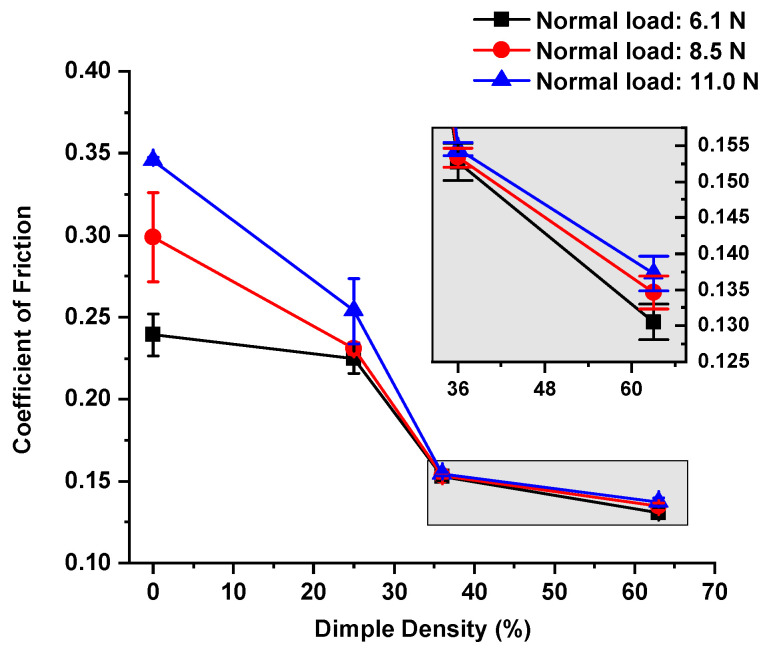
Comparison of CoFs in the non-textured case and textured cases under dry conditions and normal loads of 6.1 N, 8.5 N, and 11.0 N.

**Figure 5:**
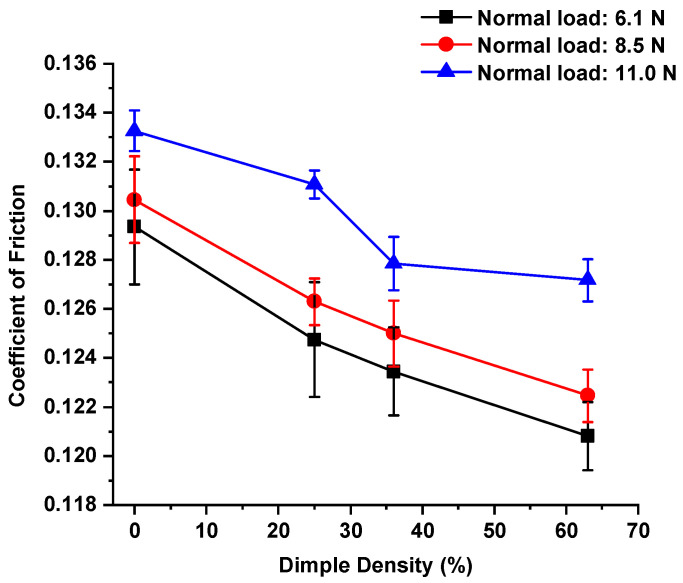
Comparison of CoFs in the non-textured case and textured cases under lubricated conditions and normal loads 6.1 N, 8.5 N, and 11.0 N.

The homemade friction tester allowed a normal load to be applied using a standard weight. The sliding speed was 10 mm/s, the sliding distance was 15 mm, and the sliding direction was perpendicular to the texture belts. The tester was calibrated before performing the test. A non-textured surface sample was prepared and tested as a reference. The average CoFs were obtained from 200 rounds of sliding under normal loads of 6.1 N, 8.5 N, and 11.0 N. The test results are presented in Figure 4 and Figure 5 for the dry and lubricated tests, respectively. Zero percent on the X-axis indicated the non-textured case as a reference.

Under dry conditions (Figure 4), the micro-dimpled texture significantly reduced the CoF compared to that observed in the non-textured case, especially for a normal load of 11.0 N. The maximum friction reduction for normal loads of 6.1 N, 8.5 N and 11.0 N was 45.5%, 54.9%, and 60.3%, respectively. For medium (36.2%) and high (63.0%) densities, the CoF error bars corresponded to the overlap of different normal loads. Thus, the differences in the average CoF obtained from varying normal loads were minimized when higher dimple density was applied.

Under lubricated conditions (Figure 5), in general, the average CoF continuously decreased as dimple density increased for each normal load, especially for 6.1 N and 8.5 N. The maximum friction reduction for normal loads of 6.1 N, 8.5 N, and 11.0 N was 6.6%, 6.1%, and 4.6%, respectively. The degree of CoF reduction for normal loads of 6.1 N and 8.5 N was only slightly different.

## 4. Contact Condition

Contact surfaces are never “flat” when they come into contact; their roughness often causes the contact areas to be extremely small, which leads to high contact pressures and stresses, resulting in failure in the contact regions [29]. Contact mechanics between solid surfaces has been previously studied [30,31], and the literature concludes that the forces and deformations around a contact area between two pressed bodies play an important role in tribological interactions. Due to the limited range of speeds and loads that our tribometer could achieve, it was not possible to evaluate a full lubrication regime. However, considering the relatively slow speed and loads used in this study, the lubrication mechanism was in the boundary regime for the lubricated case. To verify this, the contact stresses could be calculated using the Hertzian contact equations (Equations (1) and (2)) [32]. The radius of the sphere of contact *a* was related to the indenter applied normal force *F_N_*, the indenter radius *R*, and the elastic properties of the materials:

(1) $$a={\left(\frac{3\cdot F_{N}\cdot R}{4\cdot E^{*}}\right)}^{\frac{1}{3}}$$

(2) $$E^{*}={\left(\frac{1-v_{1}^{2}}{E_{1}}+\frac{1-v_{2}^{2}}{E_{2}}\right)}^{-1}$$

where *E*^∗^ is the reduced Young’s modulus and *E*_1_, *υ*_1_ and *E*_2_, *υ*_2_ describe the elastic modulus and Poisson’s ratio of the indenter and the specimen, respectively. The materials used in the experiments were AISI/ASTM 52100 bearing steel (workpiece) and a half-sphere sapphire (counter surface), and the material properties are listed in Table 2. The half-sphere sapphire was 6 mm in diameter. The maximum Hertzian contact pressure *P_max_* was given by the indenter load divided by the projected contact area:

(3) $$P_{max}=\frac{3F_{N}}{2\pi a^{2}}$$

Table 3 shows the results of Hertzian contact analysis. The values obtained using Hertzian contact theory, used to estimate the maximum pressure beneath the sapphire sphere, ranged from 1390 to 1692 MPa during friction measurements. The contact radius varied from 46 µm to 56 µm, which was smaller than the width of the micro dimples. The yield stress of AISI/ASTM 52100 was 1410 MPa [33].

Surfaces begin to deform plastically when the magnitude of the mean contact pressure reaches values above 10% of the yield stress of the surface material that has the lowest hardness [30]. For applied loads of 8.5 N and 11 N, the resultant stresses, approximated from Equation (3), were higher than the yield stress of the steel workpiece. However, the shearing action of the tip had not been considered. This analysis indicated that the contact area was under severe pressure, which caused plastic deformation of the steel workpiece. Consequently, it was highly probable that the lubricated tests were performed under the boundary lubrication regime, as predicted, because of the stresses and friction coefficients obtained during the reciprocation tests.

## 5. Discussion

**Figure 6:**
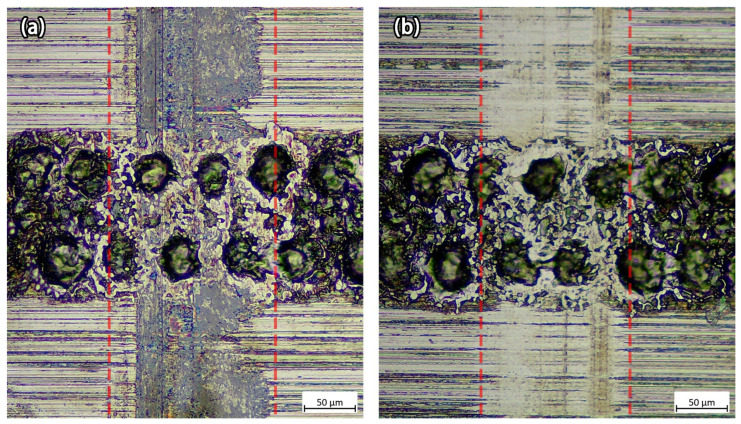
The textured workpiece after the sliding tests for both (**a**) dry and (**b**) lubricated conditions (normal load: 11.0 N; dimple density: 63%; magnification: 20×).

The sliding test results suggested that the proposed micro-dimpled texture reduced friction under both lubricated and dry conditions using a relatively low sliding speed, particularly for dry conditions. The mechanism that allowed the dimples to reduce friction still needs to be discussed. Figure 6 shows the textured workpiece after the sliding tests with a normal load of 11.0 N, in dry and lubricated conditions. The plastic deformation and wear marks were clearly visible. After 200 rounds of sliding, the basic surface profile of the dimples was retained, but the surface bulges around the dimples were worn down. According to the Bowden and Tabor theory [34,35], the friction force *F_S_* under lubricating conditions is expressed as follows:

(4) $$F_{S}=A_{r}\left\{\alpha s_{m}+\left(1-\alpha \right)s_{i}\right\}$$

For dry conditions, the friction force *F_S_* is as follows:

(5) $$F_{S}=A_{r}s_{m}$$

where *A_r_* is the real contact area; *α* is the ratio of solid contact area due to the breakage of the lubricant film; *s_m_* is the shear strength of the solid at contact; and *s_i_* is the shear strength of the lubricant film. In the sliding tests, the only variable was the micro-dimpled texture for both dry and lubricated conditions. Therefore, it was possible to infer that the smaller solid-to-solid contact area, due to the micro-dimpled texture, resulted in less friction force compared to the friction force in the non-textured case. Furthermore, the total contact area along the contact path was reduced with more dimples, which may have explained the influence of dimple density on friction reduction, as shown in Figure 4 and Figure 5. In addition, previous publications also suggested that texture could trap the micro-abrasive, further reducing friction [13,36]. That effect was not confirmed in this study, but it could still be one of the reasonable explanations for friction reduction.

The test results and surface observations of both dry and lubricated tests indicated that although the lubricant significantly minimized CoF, the dominant friction behaviour at the friction interface was still solid contact. Thus, the lubricated tests were performed under the boundary lubrication regime. There was no coherent lubricant film effect at the contact area. Simultaneously, the mechanism of the dimple effect may have been different between lubricated and dry conditions. In addition, in the sphere-flat contact, the lubricant flow domain showed a convergent shape due to the spherical shape of the sapphire and the flow space shrank gradually until reaching the contact area, which may have led to the generation of hydrodynamic pressure from the dimples or the convergent structure itself.

**Figure 7:**
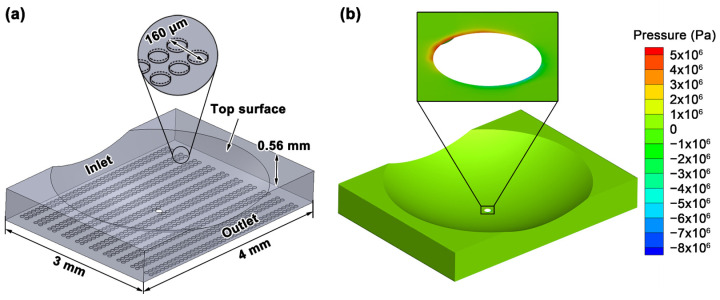
Schematic of (**a**) the flow domain of the CFD model and (**b**) the pressure distribution in the flow domain.

A computational fluid dynamics (CFD) model was designed based on the experimental setup to check the hydrodynamic pressure generation. A small flow area was chosen from the experimental setup. The flow area was 3 mm by 4 mm, as shown in Figure 7. The film thickness was set at 10 μm. The texture distribution in the model followed the experimental workpiece. The texture unit was designed based on a real dimple, which was fabricated on the workpiece, that had a diameter of 70 μm and depth of 8 μm. The width of the texture belt was 160 μm. Figure 7a shows the flow domain of the CFD model, based on a high dimple density case under a normal load of 11.0 N. The hole at the center of the flow domain indicated the contact area. The diameter of the hole was set based on the Hertzian contact calculation. In Figure 7a, the inlet and outlet indicate the fluid inflow boundary and outflow boundary of the CFD model, respectively. The inlet and outlet were defined as pressure-based; the operating pressure was set to 101,325 Pa. The bottom wall and two side walls were stationary; the translational motion was set so that the top sphere surface mimicked the relative motion of the friction pair, and the translational motion was set to 0.01 m/s. Based on the Hertz contact analysis, we assumed no coherent lubricant film was in the contact area. Thus, the contact area was excluded from the flow domain model.

An open-source CFD code OpenFOAM was used in this study. The total element number of the flow domain mesh was 9.3 × 10^6^. Due to the low sliding speed applied in the test, the cavitation was negligible in the simulation. Based on the Navier–Stokes equations, mass conservation Equation (6) and momentum conservation Equation (7) are shown as follows:

(6) ∇·v=0

(7) $$\rho \left(v\cdot \nabla \right)=-\nabla p+\mu \cdot \nabla^{2}\;v$$

where ***ν*** is the velocity vector, and ∇ is the Hamilton operator. The density of the engine oil *ρ* was 850 kg/m^3^, and the dynamic viscosity *μ* was 0.1422 kg/m·s at 25 °C. The simulation result of pressure distribution is shown in Figure 7b. No pressure buildup occurred around the dimples, but a small high-pressure zone was observed around the contact zone. The high-pressure zone implied that the convergent form in sphere-flat contact may have contributed to the lubricant film formation rather than the proposed dimples.

## 6. Conclusions

This paper presents the performance of micro-dimpled texture using varied dimple densities under different normal loads and in dry and lubricated conditions. The micro-dimpled textures were fabricated using LST. Reciprocating sliding tests were conducted to determine if the micro-dimpled texture reduced friction under dry and lubricated conditions. The following conclusions were drawn from this study:

- For the lubricated condition, CoF decreased by 45.5%, 54.9%, and 60.3% under normal loads of 6.5 N, 8.1 N, and 11 N, respectively;
- For the dry condition, CoF decreased by 4.3%, 6.1%, and 6.6% under normal loads of 6.5 N, 8.1 N, and 11 N, respectively. As the normal load increased, CoF increased in all non-textured and textured cases;
- Friction reduction is proportional to texture density. CoF showed a linear decrease as micro dimple density increased under dry and lubricated conditions;
- According to surface observations and the simulation, the dominant mechanism of the micro-dimpled effect on friction reduction could be the reduced contact area.
- For the friction reduction, the contribution of hydrodynamic pressure generation due to the micro-dimpled texture under lubricated conditions could be neglected according to the simulation result. Future surface texture design under high-pressure contact conditions could focus on the contact area rather than the lubricant flow mechanism.

## Figures and Tables

**Table 1:** Specifications of the laser marking system.

Specifications	Target
Laser type	Diode laser (Raycus™ RFL-P20Q)
Output power	0–20 W
Wavelength	1060 nm
Pulse frequency	20–200 kHz
Spot size	50 μm
Spot moving speed	0–7000 mm/s

**Table 2:** Physical properties of sapphire (Al_2_O_3_) and AISI 52100 steel at room temperature.

Properties	Sapphire	AISI 52100 Steel
Density *ρ*	3910 kg/m^3^	7810 kg/m^3^
Young’s modulus *E*	345 GPa	200 GPa
Poisson’s ratio *υ*	0.29	0.3
Hardness, Vickers *H*	2300	700

**Table 3:** Contact pressures calculated using Hertzian analysis.

Normal Force *F_N_*	6.1 N	8.5 N	11.0 N
Contact circular radius *a*	46 µm	51 µm	56 µm
Contact area	0.0079 mm^2^	0.0082 mm^2^	0.0098 mm^2^
Max. Hertzian contact pressure *P_max_*	1390 MPa	1553 MPa	1692 MPa
